# Investigating Intranasal Olfactory Mucosa Progenitor Cells Administration in Compensating Hippocampal Atrophy in Alzheimer Disease: A Novel Perspective

**DOI:** 10.1002/alz.088212

**Published:** 2025-01-03

**Authors:** Mohammad Walid, Youssef A. Ismail, Youssef M. Abd El‐Satar, Hazim Mohamed, Youssef Haitham

**Affiliations:** ^1^ STEM Neurology & Neuropsychological0 Research Group Egypt (SNRGE), Port Said, Port Said Egypt; ^2^ Faculty of Medicine, Arish University, Arish, North Sinai Egypt; ^3^ Faculty of Medicine Port Said Univeristy, Egypt, Port Said, Port Said Egypt; ^4^ Faculty of Medicine Cairo Univeristy, Egypt, Cairo, Cairo Egypt; ^5^ Faculty of Medicine Helwan University, Cairo, Helwan Egypt; ^6^ Faculty of Medicine Suez Canal Univeristy, Egypt, Ismailia, Ismailia Egypt

## Abstract

**Background:**

The olfactory mucosa cells are capable of lifelong neurogenesis providing a viable source of progenitor cells. Olfactory mucosa progenitor cells (OMPCs) have alleviated several cerebral ischemia/reperfusion damage markers. OMPCs are safely obtainable from the upper nasal cavity. The aim is to investigate the potential of administering the OMPCs to achieve neurogenesis in regions of hippocampal atrophy in Alzheimer’s disease (AD)

**Method:**

We reviewed database searches through December 2023 in MEDLINE, PsycINFO, and the Cochrane Central Register of Controlled Trials, and additional searches for ongoing trials through ClinicalTrials.gov and Current Controlled Trials (ISRCTN Register).

**Result:**

Based on a trial conducted to assess the homing capacity of human OPMCs applied intranasally, the OPMCs migrated to the sites of soma atrophy and axonal swelling. Mapping the position of the applied cells, the mean distance (+SD) from OMPCs to regions of axonal damage indicated *B‐APP^+^* (the same marker in CA1 hippocampal atrophy in AD) was 71.8+60.3 µm; OMPCs to atrophic cell bodies indicated *p‐c‐jun^+^* (the same marker in CA2 hippocampal atrophy in AD) was 188.7 + 320 µm; OMPCs to random sites was 3929.3 + 2636.3 µm. Another trial was conducted on mice to confirm whether the OMPCs also exhibit high capacity of neurogenesis. The presence of relay neurons derived from the OMPCs differentiation was evident and traced by the Wheat germ agglutinin (WGA) expression in the injured regions in the spinal cords of the subjects. Based on the analysis of evidence and reviewing the literature related to the pathogenesis and markers of AD hippocampal atrophy, human OMPCs intranasal administration have high homing capacity which make them have a strong potential to safely migrate to the site of axonal damage (*B‐APP* ^+^ region) and sites of soma atrophy (*p‐c‐jun^+^* region) in hippocampus of AD patients. At the site of homing, OMPCs differentiate into trans‐synaptic relay neurons, expressing WGA protein, which reconstructs the neural pathways in the site of atrophy not only by acting as a scaffold, but also by differentiating into relay‐neurons

**Conclusion:**

OMPCs intranasal administration is a novel technique which may have potential to restore neural pathways in subjects with AD presenting with hippocampal atrophy.

